# Detection of germline variants in Brazilian breast cancer patients using multigene panel testing

**DOI:** 10.1038/s41598-022-07383-1

**Published:** 2022-03-09

**Authors:** Rodrigo Santa Cruz Guindalini, Danilo Vilela Viana, João Paulo Fumio Whitaker Kitajima, Vinícius Marques Rocha, Rossana Verónica Mendoza López, Yonglan Zheng, Érika Freitas, Fabiola Paoli Mendes Monteiro, André Valim, David Schlesinger, Fernando Kok, Olufunmilayo I. Olopade, Maria Aparecida Azevedo Koike Folgueira

**Affiliations:** 1 Centro de Investigação Translacional em Oncologia (CTO), Instituto do Cancer do Estado de Sao Paulo (ICESP), Hospital das Clínicas da Faculdade de Medicina da Universidade de Sao Paulo (HCFMUSP), Av Dr Arnaldo, 251, 8th floor, Cerqueira Cesar, São Paulo, SP ZIP 01246‑000 Brazil; 2 Instituto D’or de Pesquisa E Ensino (IDOR), Salvador, Brazil; 3 Mendelics Análise Genomica SA, Sao Paulo, Brazil; 4 Department of Medicine, Center for Clinical Cancer Genetics and Global Health, The University of Chicago, Chicago, IL 60637 USA

**Keywords:** Cancer genomics, Cancer screening

## Abstract

Genetic diversity of germline variants in breast cancer (BC) predisposition genes is unexplored in miscegenated populations, such those living in Latin America. We evaluated 1663 Brazilian BC patients, who underwent hereditary multigene panel testing (20–38 cancer susceptibility genes), to determine the spectrum and prevalence of pathogenic/likely pathogenic (P/LP) variants and variants of uncertain significance (VUS). Associations between P/LP variants and BC risk were estimated in a case–control analysis of BC patients and 18,919 Brazilian reference controls (RC). In total, 335 (20.1%) participants carried germline P/LP variants: 167 (10.0%) in *BRCA1/2*, 122 (7.3%) in BC actionable non-BRCA genes and 47 (2.8%) in candidate genes or other cancer predisposition genes. Overall, 354 distinctive P/LP variants were identified in 23 genes. The most commonly mutated genes were: *BRCA1* (27.4%), *BRCA2* (20.3%), *TP53* (10.5%), monoallelic *MUTYH* (9.9%), *ATM* (8.8%), *CHEK2* (6.2%) and *PALB2* (5.1%). The Brazilian variant *TP53* R337H (c.1010G>A, p.Arg337His), detected in 1.6% of BC patients and 0.1% of RC, was strongly associated with risk of BC, OR = 17.4 (95% CI: 9.4–32.1; p < 0.0001); monoallelic *MUTYH* variants c.1187G>A and c.536A>G, detected in 1.2% (0.9% RC) and 0.8% (0.4% RC) of the patients, respectively, were not associated with the odds of BC, the former with OR = 1.4 (95% CI: 0.8–2.4; p = 0.29) and the latter with OR = 1.9 (95% CI: 0.9–3.9; p = 0.09). The overall VUS rate was 46.1% for the entire patient population. Concluding, the use of multigene panel testing almost doubled the identification of germline P/LP variants in clinically actionable predisposition genes in BC patients. In Brazil, special attention should be given to *TP53* P/LP variants.

## Introduction

Breast cancer (BC) is the most common cancer in women worldwide. In Brazil, an average of 66,280 women are diagnosed with carcinoma of the breast every year, accounting for 29.7% of all cancers in the female population^1^. Inherited pathogenic/likely pathogenic variants (P/LP) in highly penetrant predisposition genes are thought to be involved in about 10% of BC cases. Among the hereditary forms, the most frequent events are germline P/LP variants in *BRCA1*/*2* genes which predispose to hereditary breast and ovarian cancer syndrome (HBOC). The prevalence and spectrum of *BRCA1/2* P/LP variants vary among different populations and are responsible for only approximately 25–50% of the familial risk of BC^2–4^. As DNA sequencing technologies evolved, other cancer susceptibility genes have been discovered, including high-penetrant genes such as *TP53*, *CDH1*, *STK11*, *PTEN* and *PALB2* (>  4 fold cancer relative risk), moderate-penetrant genes such as *CHEK2* and *ATM* (1.5–4 fold cancer relative risk), and a number of common low-penetrant BC susceptibility loci identified through genome-wide association studies (1–1.5 fold cancer relative risk)^5–7^. The mutational spectrum of germline mutations in BC predisposition genes have been reported in single populations, with the majority of reports focused on Caucasians from Europe and North America. The population from Southern Hemisphere countries, except for Australia, are underrepresented and understudied in cancer genetic epidemiology research^2^.

The Brazilian population has unique ethnic characteristics. People miscegenation is a universal phenomenon, due to globalization and large waves of immigration. Brazil is considered an ethnic “melting pot”, reflecting an admixture of European, Native American and Sub-Saharan African people, in addition to immigrants from a large number of European, Asian and Middle Eastern countries. Hence, Brazilian people offer a unique opportunity to advance the understanding of cancer genetic features in a miscegenated population^8^.

In Brazil, the majority of the inherited BC studies focused on the analyses of *BRCA1/2* as well as *TP53*, given the relatively high population frequency of the *TP53* R337H (also known as, c.1010G>A, p.Arg337His) variant in people from the South and Southeast regions of Brazil^9^. However, the likelihood of carrying P/LP variants in other BC susceptibility genes among *BRCA1/2* and *TP53*-negative patients is largely unexplored.

Recent advances in next generation sequencing (NGS) technology has reduced the cost of massively parallel sequencing, provided to physicians and patients the option of sequencing multiple genes simultaneously and broadened our understanding of the genetic etiology of inherited cancers. Multigene panel testing has proved useful as a diagnostic tool for disorders where similar phenotypes can be influenced by multiple genes such as hereditary predisposition to BC, uncovering potentially actionable findings that may be missed by traditional testing paradigms. Several laboratories have released commercial multigene panel testing ranging from six to > 100 genes^10^. Panels are cheaper, faster and increase the yield of genetic findings, more than doubling the mutation detection rate in *BRCA1/2*-negative patients with suspected HBOC^11–18^. However, finding a mutation in a gene where the cancer risks and/or management strategies are not known, as well as the identification of higher numbers of variants of uncertain significance (VUS), can make the results cumbersome and challenging for a physician to interpret and guide treatment^10^.

Panels have been widely available in Brazil within the past 7 years, but no study has yet assessed the prevalence and mutational spectrum of germline variants in BC susceptibility genes other than *BRCA1/2* and *TP53* in a large cohort of individuals with BC, who were referred for genetic evaluation. Given the rapid uptake of multigene panel testing in clinical practice, these data are urgently needed to inform genetic counseling. In this study, we report the results from 1663 consecutive individuals with a history of BC who were referred for multigene panel testing.

## Results

### Study population and prevalence of P and LP variants

This study involved a nationwide sample of 1663 consecutive BC patients who underwent germline genetic testing with a multigene cancer panel between 2015 and 2017. Over half of the tests (or 51.9%) were from patients who inhabited the Southeast region of Brazil. Patients from all other regions were also well represented, except for patients from the North region. This information appears on Table 1.

**Table 1:** Number of carriers of P/LP germline variants according to age and living country region.

Patients with a positive finding: n (%)									
	Total cohort 1663 (100%)	BRCA1 97 (5.8%)	BRCA2 72 (4.3%)	BRCA1/2 167 (10.0%)	TP53 37 (2.2%)	TP53 R337H 26 (1.6%)	High-penetrant BC genes 223 (13.4%)	Moderate- penetrant BC genes 69 (4.1%)	Multigene panel 335 (20.1%)
**Age at BC diagnosis or age at testing**									
≤ 35 years	481	45 (9.3)	22 (4.6)	66 (13.7)	16 (3.3)	7 (1.4)	86 (17.9)	23 (4.8)	124 (25.8)
≤ 50 years	1289	88 (6.8)	57 (4.4)	143 (11.1)	33 (2.6)	22 (1.7)	192 (14.9)	56 (4.3)	286 (22.2)
≤ 65 years	1593	96 (6.0)	69 (4.3)	163 (10.2)	37 (2.3)	26 (1.6)	219 (13.7)	65 (4.1)	326 (20.5)
Mean, y (SD)	42.9 (11.2)	38 (9.2)	42.1 (10.6)	39.8 (10)	39.2 (10.5)	42.2 (10.9)	39.9 (9.9)	42.6 (12.1)	40.6 (10.6)
Median, y(min–max)	41 (12–87)	36 (23–66)	40 (21–76)	38 (21–76)	38 (21–65)	40.5 (23–65)	39 (21–76)	39 (24–87)	39 (21–87)
**Regions of Brazil**									
Southeast	863	54 (6.3)	44 (5.1)	96 (11.1)	22 (2.5)	15 (1.7)	130 (15.1)	37 (4.3)	190 (22.0)
South	293	15 (5.1)	12 (4.1)	27 (9.2)	10 (3.4)	8 (2.7)	40 (13.6)	11 (3.7)	60 (20.5)
North	26	0 (0.0)	0 (0.0)	0 (0.0)	0 (0.0)	0 (0.0)	0 (0.0)	3 (11.5)	3 (11.5)
Northeast	283	9 (3.2)	7 (2.5)	16 (5.6)	2 (0.7)	1 (0.3)	21 (7.4)	11 (3.9)	40 (14.1)
Central-west	198	19 (9.6)	9 (4.5)	28 (14.1)	3 (1.5)	2 (1.0)	32 (16.2)	7 (3.5)	42 (21.2)

High-penetrant genes: *BRCA1*, *BRCA2*, *CDH1*, *NF1*, *PALB2*, *PTEN*, *STK11* and *TP53*. Moderate-penetrant genes: *ATM*, *BARD1*, *BRIP1*, *CHEK2*, *RAD51C* and *RAD51D*. Positive findings: carriers of likely pathogenic and pathogenic variants.

*BC* breast cancer, *SD* standard deviation, *y* years.

Among all patients, mean age at BC diagnosis was 42.9 ± 11.2 years and median age was 41 years (min: 12 years–max: 87 years) (Table 1). Almost all, or 1650 (99.2%) patients were women. There was a significant age difference between sexes (women: 42.7 ± 11.1 years vs men: 61.1 ± 10.5 years; p < 0.001).

Among all 1,663 patients, 335 or 20.1% carried a P/LP variant in at least one gene (Table 1); among patients aged ≤ 35 years, 25.8% carried a P/LP variant, significantly more than in the whole cohort (25.8% vs. 20.1%; OR = 1.3; 95% CI: 1.0–1.6; p < 0.04).

**Figure 1:**
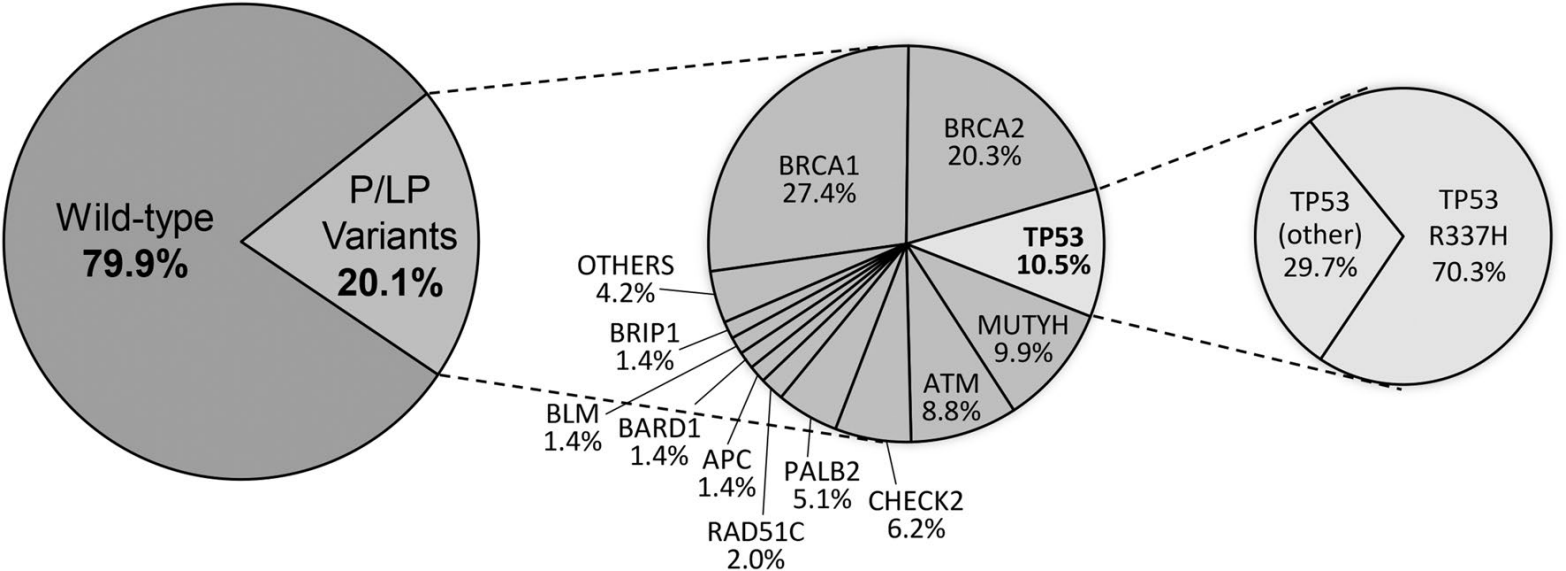
Contribution of *TP53* mutation in Brazilian breast cancer patients (n = 1663).

Overall, 335 (20.1%) participants carried germline P/LP variants, including 223 (13.4%) in high-penetrant BC genes [*BRCA1* 97 (5.8%), *BRCA2* 72 (4.3%), *TP53* 37 (2.2%), *PALB2* 18 (1.1%), *CDH1* 1 (0.1%), *NF1* 1 (0.1%), *PTEN* 1 (0.1%)] and 69 (4.1%) in moderate-penetrant BC genes [*ATM* 31 (1.9%), *CHEK2* 22 (1.3%), *RAD51C* 7 (0.4%), *BRIP1* 5 (0.3%), *BARD1* 1 (0.1%), *RAD51D* 1 (0.1%)]. Of note, 56 (3.4%) patients had a P/LP variant in candidate genes or genes traditionally associated with other hereditary cancers: *MUTYH* (n = 35), *APC* (n = 5), *BLM* (n = 5), *FANCC* (n = 3), *PMS2* (n = 2), *RECQL* (n = 2), *MEN1* (n = 1), *MSH2* (n = 1), *MLH1* (n = 1). All these P/LP variants are shown in Fig. 1 and Table 2. Among mutation carriers, there were eight patients carrying exonic deletions, including: *BRCA1* (2), *MUTYH* (2), *ATM* (1), *BRCA* (1), *MLH1* (1), *RAD51C* (1); and five presenting Alu insertions in *BRCA2*. These alterations are listed in Table 2.

**Table 2:** Pathogenic and likely pathogenic variants per gene in a cohort of breast cancer patients submitted to multigene panel testing [Bold: variants detected in more than one patient, (n)].

Gene	Pathogenic variants and rearrangements	Likely Pathogenic variants and rearrangements	Total
APC		**c.3920T>A (4)**; c.6287C>G	5
ATM	c.1495C>T; c.2413C>T; c.3272_3273delAG; c.3576G>A, **c.3802delG (4)**; c.6404dupT; **c.640delT (3)**; c.748C>T; c.7630-2A>C; c.7789-3T>G; **c.7875_7876delTGinsGC (2)**; c.7876G>C; c.7886_7890delTATTA; c.8264_8268delATAAG; c.8292_8293delTG; c.8494C>T; c.9022C>T; Del. ex. 27–28	c.1065 + 1G>T; c.6348-1G>A; **c.6975G>A (3)**; c.9023G>A; c.9146delT	31
BARD1	**c.176_177delAG (4)**	c.1758delT	5
BLM	**c.1642C>T (2)**; c.2207_2209delATC	c.2663-2A>G; c.3875-2A>G	5
BRCA1	c.1A>G; c.1088delA; c.1340_1341insG; c.1380_1381insT; c.1492delC; **c.1687C>T (5)**; **c.181T>G (2)**; **c.188T>A (2)**; c.1962dupG; c.2037_2038insC; **c.211A**>**G (3)**; c.2176_2177delCT; c.2217dupA; **c.2405_2406delTG (2)**; c.2889_2890delTG; **c.2960dupA (2)**; c.3257T>G; **c.3331_3334delCAAG (13)**; **c.3481_3491delGAAGATACTAG (2)**; c.3598C>T; c.3817C>T; c.3916_3917delTT; c.4065_4068delTCAA; c.4183C>T; c.4357 + 1G>T; **c.4484G>T (2)**; c.4675 + 1G>A; c.470_471delCT; c.5030_5033delCTAA; c.5062_5064delGTT; **c.5074 + 2T>C (3)**; c.5096G>A; **c.5266dupC (28)**; c.5444G > A; c.66dupA; c.679G>T; **c.68_69delAG (2)**; Del. ex. 1	c.192T >G; c.3G>A; c.4964C>T; c.5165C>T; Del. ex. 1–11	97
BRCA2	c.1813delA; **c.1813dupA (3)**; c.2376C>G; **c.2808_2811delACAA (5)**; c.289G>T; **c.2T>G(3)**; c.3264dupT; c.4380_4381delTT; c.466_467delGA; c.517-1G>A; c.5216dupA; c.5303_5304delTT; c.5616_5620delAGTAA; c.5720_5723delCTCT; c.6037A>T; c.631G>A; **c.6405_6409delCTTAA(5)**; c.6450dupA; c.6468_6469delTC; c.6591_6592delTG; **c.6656C**>**G (4)**; c.7060C>T; c.7191dupT; c.738delT; c.7617 + 1G>A; **c.793 + 1G>A (2)**; c.8067T>A; c.8174G>A; c.8243G>A; **c.8488-1G>A (5)**; c.8488-2A>C; c.9041C>G; c.9097delA; c.9097dupA; c.9154C>T; **c.9382C>T (2)**; c.93G>A; c.2498_2506delinsAACAG; **c.156_157insAlu (5)**	c.2167_2168delAG; c.425G>T; c.4963delT; c.6039delA; c.6290_6291insTA; c.8755-1G>A; c.9371A>T; Del. ex. 2	72
BRIP1	c.2392C>T	c.1936-2A>C; c.1941G>C; c.205 + 1delG; c.3260dupA	5
CDH1		c.48 + 1G>A	1
CHEK2	**c.1100delC (2)**; c.1283C>T; **c.433C>T (2)**	c.1361_1362delAA; **c.1427C>T (3)**; **c.319 + 2T>A (2)**; **c.349A>G (7)**; **c.470T>C (4)**	22
FANCC	c.1393C>T; **c.456 + 4A**>**T (2)**		3
MEN1		c.1132C>T	1
MLH1	Del. ex. 17 to 19		1
MSH2	c.1147C>T		1
MUTYH	c.1147delC; **c.1187G>A (13)**; c.1437_1439delGGA; c.325C>T; **c.389-1G>C (2)**; **c.536A>G (8)**; c.545G>A; **Del. ex 4–16 (2)**	**c.736G>T (2)**; **c.934-2A>G (4)**	35
NBN	c.156_157delTT		1
NF1		c.2251G>C	1
PALB2	**c.1042C>T (2)**; c.1140_1143delTCTT; c.1240C>T; c.1424dupC; c.1539dupA; c.3008delA; c.3027delT; c.509_510delGA; **c.50T**>**G (2)**; c.715delA	c.108 + 1G>A; c.1671_1674delTATT; c.2587-1G>C; **c.3271C>T (2)**; c.3350G>A	18
PMS2		**c.137G>T (2)**	2
PTEN		c.209 + 2T>C	1
RAD51C	c.709C>T; **c.890_899delTTGTTCCTGC (2)**	c.404G>A; **c.656T>C (2)**; Del. ex. 4	7
RAD51D	c.694C>T		1
RECQL		c.493_497delAGTTC; c.675_676insGATGTAG	2
TP53	**c.1010G>A (26)**; c.257_279delCACCAGCCCCCTCCTGGCCCCTG; **c.733G>A (2)**; c.742C>T; **c.743G>A (2)**; c.818G>A; c.844C>T	c.396G>C; c.718A>G; c.845G>C	37

Eighteen patients carried P/LP variants in two genes and one patient in three different genes. Of note, two patients presented P/LP variants in both *BRCA1* and *BRCA2*, three patients in *TP53* R337H in association with *BRCA1* c.5266dupC or monoallelic *MUTYH* (n = 2). Additionally, mutated monoallelic *MUTYH*, particularly *MUTYH* c.1187G>A, was the most frequent partner of other mutated genes (such as, *BRCA1*, *BRCA2*, *PALB2* and *TP53*), detected in seven patients (Supplementary Table S1).

*MUTYH* P/LP variants were detected in 2.1% of the patients, including monoallelic *MUTYH* c.1187G>A, which was detected in 13 out of 1,068 BC patients, as well as in 170 out of 18,919 reference controls (1.2% vs 0.9%; OR = 1.4; 95% CI: 0.8–2.4; p = 0.29) and *MUTYH* c.536A>G, detected in 8 patients and 76 reference controls (0.8% vs 0.4%; OR = 1.9; 95% CI: 0.9–3.9; p = 0.09).

Age at BC diagnosis was significantly lower for *BRCA1* P/LP variant carriers (38.0 ± 9.2 years) than in patients who were not P/LP germline carriers (43.5 ± 11.3; p < 0.001). Age at diagnosis was not associated with carriers of P/LP variants in any other genes when compared with non-carriers. Among 19 patients older than 75 years, four were P/LP variant carriers (21%), one in *ATM*, one in *BRCA2* and two in *CHEK2*. Among 13 male patients, two (15.4%) were P/LP variant carriers, both in *BRCA2*.

### Mutation spectrum of P and LP variants

**Figure 2:**
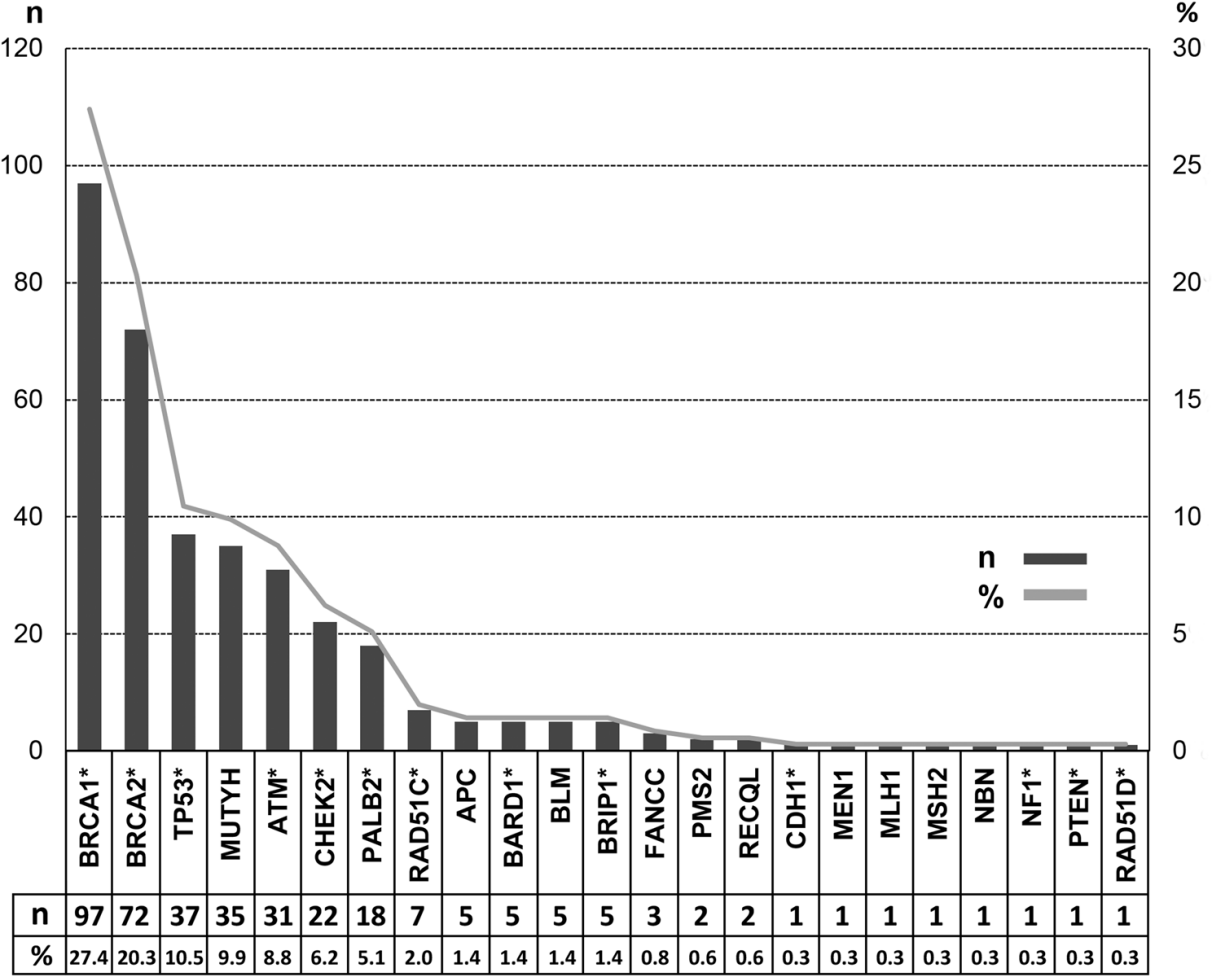
Mutation spectrum of pathogenic and likely pathogenic variants. *Clinically actionable breast cancer genes.

Overall, 354 P/LP variants were identified in 335 patients. Among these P/LP variants (100%), the three most frequently mutated genes were *BRCA1* (27.4%), *BRCA2* (20.3%) and *TP53* (10.5%), followed by *MUTYH* (9.9%), *ATM* (8.8%), *CHEK2* (6.2%) and *PALB2* (5.1%), as shown in Fig. 2.

Allelic heterogeneity among the patients was reflected in the appearance of 188 distinct P/LP variants in 23 genes (Supplementary Table S2). Although the mutational profile was heterogeneous, recurrent variants (detected in three or more individuals) were found in 8 genes: *APC* c.3920T>A; *ATM* c.3802delG, c.640delT and c.6975G>A; *BARD1* c.176_177delAG; *BRCA1* c.5266dupC, 3331_3334delCAAG, c.1687C>T and c.211A>G; *BRCA2* c.6405_6409delCTTAA, c.156_157insAlu, c.2808_2811delACAA, c.8488-1G>A, c.6656C>G, c.1813dupA and c.2T>G; *CHEK2* c.349A>G, c.470T>C and c.1427C>T; *MUTYH* c.1187G>A, c.536A>G and c.934-2A>G; and *TP53* c.1010G>A (Table 2). The most prevalent *BRCA1* recurrent variants, which were the European founder variants c.5266dupC (n = 28) and c.3331_3334delCAAG (n = 13), accounted for 43.2% of all *BRCA1* reported variants. The European founder *CHEK2* recurrent variant c.349A>G (n = 7) accounted for 41.2% of all *CHEK2* reported variants.

The *TP53* R337H is of particular interest because it is widespread in Brazil due to a founder effect and is present in 0.3% of the southern and southeastern general populations^20^.

### The Brazilian TP53 R337H variant

Overall, *TP53* was the third most frequently mutated gene and contributed to 2.2% of BC cases in our cohort. *TP53* P/LP variants were detected in 37 out of 1,663 BC patients and in 21 out of 18,919 reference controls (2.2% vs 0.1%; OR = 20.5; 95% CI: 11.6 – 39.9; p < 0.001). It is noteworthy that the *TP53* variants were concentrated in the South and Southeast (86.5%; Table 1) compared to the other regions of Brazil (32 vs 5; OR = 2.9; 95% CI: 1.1–7.4; p = 0.03).

The Brazilian *TP53* R337H variant accounted for 70.3% of all *TP53* reported P/LP variants and was also concentrated in patients from the South and Southeast regions of Brazil (Table 1). This variant was detected in 26 out of 1,663 BC patients, as well as in 17 out of 18,919 reference controls (1.6% vs 0.1%; OR = 17.4; 95% CI: 9.4 – 32.1; p < 0.0001) . Another 10 patients had mutations in the *TP53* DNA binding domain. *TP53* R337H carriers were diagnosed with BC an average of 10 years older than patients who carried *TP53* pathogenic variants within typical DNA-binding domain (42.2 ± 10.9 years vs. 32.3 ± 5.1 years, p < 0.007).

### VUS in Brazilian patients with BC

**Figure 3:**
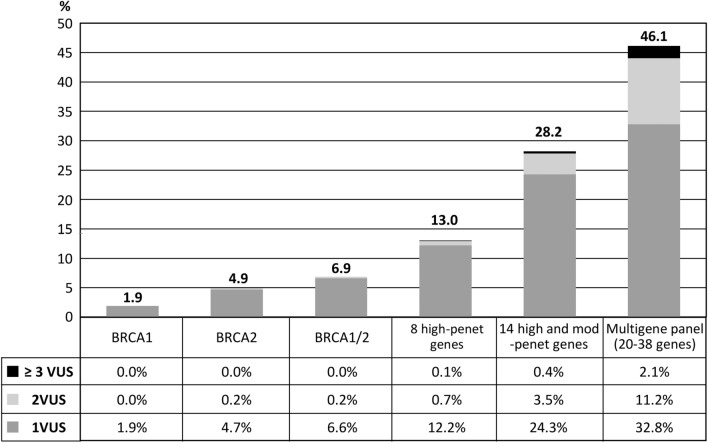
Frequency of variants of unknown significance (VUS). Cumulative fraction of clinical cases with one or more VUS, irrespective of pathogenic variants observed, as the scope of testing increases. High-penetrant genes: *BRCA1*, *BRCA2*, *CDH1*, *NF1*, *PALB2*, *PTEN*, *STK11* and *TP53*; moderate-penetrant genes: *ATM*, *BARD1*, *BRIP1*, *CHEK2*, *RAD51C* and *RAD51D*.

**Figure 4:**
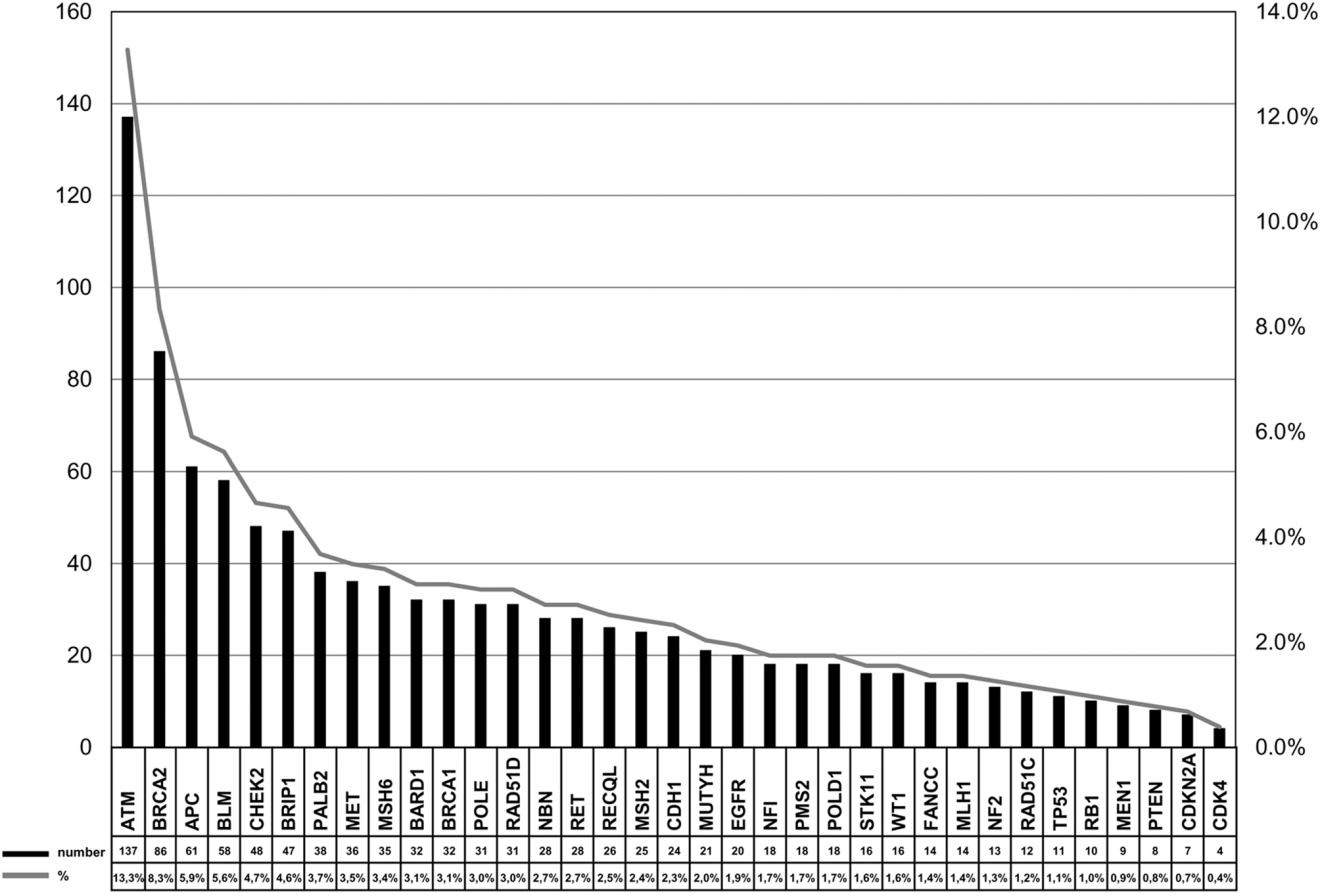
Number and percentage of variants of unknown significance per gene.

The overall VUS rate was 46.1% for the entire patient population, with 13.4% having two or more VUS (Fig. 3). As expected, the prevalence of VUS increased considerably with the number of genes tested. The chance to detect a VUS was 6.7% if only the *BRCA1/2* genes were tested. Comparing to a *BRCA1/2* test, this chance was approximately 2 times higher if the 8 high-penetrant BC genes were tested (OR = 2.0; 95% CI: 1.6–2.6; p < 0.0001), 5 times higher if the 14 high/moderate-penetrant BC genes were tested (OR = 5.3; 95% CI: 4.2–6.6; p < 0.0001), and almost 12 times higher if a multigene panel (20–38 genes) was tested (OR = 11.6; 95% CI: 9.4–14.4; p < 0.0001). Among all the genes tested, the highest number of VUS was detected in *ATM*, followed by *BRCA2* (Fig. 4). Approximately 90% of the VUS were missense variants (Supplementary Table S3).

## Discussion

This is the largest nationwide cohort of Brazilian BC patients who underwent NGS mutigene panel testing reported to date. In this study, both allelic heterogeneity and founder mutations played a role in inherited BC. The most commonly mutated genes were *BRCA1/2*, which were identified in 10% of the entire cohort and accounted for almost 50% of all P/LP germline variants identified. In accordance with previous research from different countries, the use of a multigene panel test doubled the yield of P/LP variants detected, as well as increased in 12 times the chance of finding a VUS. Most significantly, this study differs from the others because it highlights the important contribution of Li-Fraumeni syndrome (LFS) to inherited BC burden in Brazil, due to the Brazilian *TP53* R337H variant. It is worth emphasizing that the number of patients carrying this mutation is similar to the number of patients with *BRCA1* c.5266dupC, which is the most prevalent *BRCA1* pathogenic variant in our study.

Patients from all regions of the country were represented, mainly from the Southeast region of Brazil, which is the most densely populated, with more than 89 million people (or 42% of the Brazilian population). Patients from all other regions were also well represented, except for patients from the North region, which is the least densely populated with 8.8 million people in 3.87 million km^2^, covered mostly by the Amazon Rainforest.

The estimated frequency in the general population of P/LP *BRCA1/2* mutations is 1:800–1:1000 per gene^21^; however, the prevalence of pathogenic variants in *BRCA1/2* varies considerably between different ethnic groups and geographic areas. In Brazil, there are no large population studies yet, so we do not have reliable estimates of its prevalence in this scenario. The prevalence of *BRCA1/2* pathogenic variants in unselected, under the age of 35 or classified as high-risk BC patients was estimated to be 2.3%^22^, 16.5–20.4% and 3.4–22.5%, respectively^23–32^ (Table 3). Our unselected cohort probably has the bias of comprehending mainly high-risk patients, as they were probably referred for genetic testing due to suspicion of the attending physician, identified a percentage of patients with a *BRCA1/2* mutation of approximately 10%. The two most prevalent mutations are in accordance with the largest study of the Brazilian population reported to date: *BRCA1* c.5266dupC and *BRCA1* c.3331_3334delCAAG^33,34^. The *BRCA1* c.5266dupC founder pathogenic variant is the most frequently reported in Brazil by several independent studies, but has not been observed elsewhere in South America, with the exception of an Ashkenazi community in Argentina. Notwithstanding, the *BRCA1* c.3331_3334delCAAG was identified in BC patients in Spain and Portugal, as well as in Brazil, Chile, and Colombia. Despite a significant contribution of African ancestry to the genetic pool of some of the populations of Brazil, no recurrent pathogenic variants were traced back to the African continent in our cohort^35^.

**Table 3:** Prevalence of *BRCA1/2* germline variants in HBOC patients in Brazil.

References	n	Studied population	BRCA1, n (%)	BRCA2, n (%)	BRCA1/2, n (%)	Screening methodology	BRCA1 covered region	BRCA2 covered region
Gomes et al.^22^	402	Unselected BC	6 (1.5)	3 (0.8)	9 (2.3)	OMM + DS	Partial	Partial
Carraro et al.^24^	54	BC < 35 years	7 (13)	4 (7.4)	11 (20.4)	DS	Complete	Complete
Encinas et al.^23^	79	BC < 35 years	4 (5.1)	9 (11.4)	13 (16.5)	DS + MLPA	Complete	Complete
Lourenço et al.^25^	47	High-risk BC	7 (15)	NA	7 (15)	DS	Complete	NA
Dufloth et al.^26^	31	High-risk BC	1 (3.2)	3 (9.7)	4 (12.9)	OMM + DS	Partial	Partial
Silva et al.^27^	120	High-risk BC	20 (16.7)	7 (5.8)	27 (22.5)	DS + MLPA	Complete	Complete
Esteves et al.^28^	612	High-risk (PH ± FH)	19 (2.9)	3 (0.5)	21 (3.4)	OMM	Partial	Partial
Ewald et al.^29^	137	High-risk (PH ± FH)	7 (5)	NE	7 (5)	DS	c.68_69delAG, c.5266dupC	c.5946delT
Felix et al.^30^	106	High-risk (PH ± FH)	9 (8.5)	0	9 (8.5)	DS	Complete	c.5946delT, c.156_157insAlu
Palmero et al.^31^	18	High-risk (PH ± FH)	0	1 (7.1)	1 (7.1)	OMM	Partial	Partial
Fernandes et al.^34^	349	High-risk (PH ± FH)	49 (14)	26 (7.5)	75 (21.5)	DS + MLPA	Complete	Complete
Alemar et al.^32^	418	High-risk (PH ± FH)	51 (12.2)	31 (7.4)	80 (19.1)	DS + MLPA	Complete	Complete
de Souza Timoteo et al.^60^	157	High-risk (PH ± FH)	11 (7.0)	5 (3.2)	16 (10.2)	DS	Complete	Complete
Cipriano Jr et al.^38^	44	High-risk (PH ± FH)	5 (11.4)	7 (15.9)	12 (27.3)	OMM + DS	Partial	Partial
Bandeira et al.^61^	105	High-risk (PH ± FH)	10 (9.5)	4 (3.8)	14 (13.3)	DS + MLPA	complete	Complete
da Costa E Silva Carvalho et al.^39^	95	High-risk (PH ± FH)	13 (13.7)	4 (4.2)	17 (17.9)	DS + MLPA	Complete	Complete
Nagy et al.^62^	49	High-risk PMP BC	3 (6.1)	2 (4.1)	5 (10.2)	DS + MLPA	Complete	Complete
Guindalini et al. (current study)	1663	BC referred to GT	96 (5.8)	72 (4.3)	167 (10)	DS + MLPA	Complete	Complete

*BC* breast cancer, *PH* ± *FH* personal history and / or family history of breast and / or ovary cancer, *PMP* postmenopausal, *GT* genetic testing, *MLPA* multiplex ligation- dependent probe amplification, *NE* not evaluated, *OMM* other molecular methods such as *DHPLC* denaturing high performance liquid chromatography, *HRM* high resolution melting, *PTT* protein truncation test, *SSCP* single-strand conformation polymorphism, *DS* direct sequencing like Sanger or next generation sequencing (NGS).

Pathogenic variants in the *TP53* gene are very relevant for the Brazilian population. In general, the global prevalence estimates of P/LP *TP53* variants are within the range of one carrier in 3,555–5,476 individuals^36^. In Brazil, the *TP53* R337H variant is estimated to occur in about 2.7 per 1,000 individuals born in southern Brazil^20^. In the 2000s, Brazilian researchers associated the *TP53* R337H variant, which affects the oligomerization domain, with an increased risk of developing adrenocortical carcinomas. Subsequent studies have shown that the same variant could also increase the risk of other cancers, such as BC, but the penetrance was different^37–41^. The *TP53* R337H variant confers a lifetime cancer risk by age 60 years of 80% in females and 47% in males. In comparison, in classic LFS, those with mutation located in typical DNA-binding domain, the cancer risk is 90% in women and 73% in men^42^. The reasons concerning the reduced penetrance of this variant is still controversial and usually associated with its location in the gene and biochemistry stability, which is pH dependent. A recent study showed that an extended haplotype cosegregating the *TP53* R337H and *XAF1* E134* alleles may lead to a more aggressive cancer phenotype than *TP53* R337H alone, acting as a functional modifier by attenuating the transactivation of wild-type and hypomorphic *TP53* variants, such as R337H. Carriers harboring the extended haplotype were more likely to be diagnosed with sarcomas and multiple tumors, nevertheless this association was not observed in BC patients. Further studies are needed to validate these findings and evaluate their implications on genetic counseling and clinical management of *TP53* R337H carriers^43^. BC is the most common malignancy diagnosed in LFS. In Brazil, in high-risk BC patients, the prevalence of *TP53* R337H ranged from 3.4–7.1% in the South/Southeast^44,45^ and 0.9% in the Northeast region^30,46^ (Table 4). In a cohort of 815 women affected by BC in southern Brazil who developed the disease before age 45 years, the prevalence of the *TP53* R337H variant was 12.1%^45^. In our cohort, the prevalence of all P/LP *TP53* variants was 2.2%, representing the third most commonly mutated gene among BC patients. The *TP53* R337H variant was responsible for 70.3% all *TP53* mutations identified. Excluding the *TP53* R337H variant, it becomes clear that the prevalence of other mutations in *TP53* is low in the Brazilian BC patients, approximately 0.7% in the present study, in accordance with other four Brazilian studies that analyzed the entire coding region of TP53 (Table 4), following the same pattern as the worldwide prevalence^24,40,46–48^.

**Table 4:** Prevalence of *TP53* germline variants in HBOC patients in Brazil.

Reference	n	Inclusion criteria	TP53 covered region	TP53 R337H, n (%)	TP53 mutations, n (%)	Region of Brazil
Palmero et al.^9^	750	Population screening	R337H	2 (0.3)	2 (0.3)	South
Assumpção et al.^40^	123	Unselected BC	Exon 10	3 (2.4)	3 (2.4)	Southeast
Gomes et al.^41^	390	Unselected BC	R337H	2 (0.5)	2 (0.5)	Southeast
Giacomazzi et al.^45^	815	Unselected BC	R337H	70 (8.6)	70 (8.6)	South/Southeast
Carraro et al.^24^	54	BC < 35 years	Complete gene with DS	0 (0.0)	1 (2)	Southeast
Giacomazzi et al.^45^	59	High-risk BC	R337H	2 (3.4)	2 (3.4)	South
Cury et al.^44^	28	High-risk BC	Complete gene with HRM	2 (7.1)	2 (7.1)	Southeast
Silva et al.^27^	120	High-risk BC	R337H	3 (2.5)	3 (2.5)	Southeast
Felix et al.^30^	106	High-risk BC	R337H	1 (0.9)	1 (0.9)	Northeast
da Costa E Silva Carvalho et al.^39^	94	High-risk BC	Complete gene with DS	5 (5.3)	6 (6.4)	Southeast
Bandeira et al.^62^	105	High-risk BC	Complete gene with DS	1 (0.9)	1 (0.9)	Southeast
Cipriano Jr et al.^38^	44	High-risk BC	R337H	1 (2.3)	1 (2.3)	Southeast
de Souza Timoteo et al.^60^	132	High risk BC	Complete gene with DS	0 (0.0)	0 (0.0)	Northeast
Gomes et al.^48^	126	High risk breast and ovarian cancer	Complete gene with DS	0 (0.0)	1 (0.8)	Southeast
Sandoval et al.^46^	224	High risk BC	Complete gene with DS	6 (2.7)	8 (3.6)	Central West
Guindalini et al. (current study)	1663	BC refered to GT	Complete gene with DS	26 (1.6)	37 (2.2)	All

*BC* breast cancer, *DS* direct sequencing like Sanger or next generation sequencing (NGS), *GT* genetic testing, *HRM* high resolution melting.

Of note, it should also be emphasized that almost 30% of *TP53* P/LP variants occurred on sites other than R337H. Some of these pathogenic variants were already reported in Brazilian BC patients, such as c.733G>A^46^ and c.818G>A^39^, while one was detected in patients with Spanish ancestry (c.743G>A)^49^.

Thus, these results confirm that inheritance of *TP53* R337H contribute to a significant number of BC cases in Brazil. These findings reaffirm the need for differentiated guidelines for monitoring and risk reduction strategies in patients with hereditary BC in Brazil. The investigation of the *TP53* R337H variant in the Brazilian pre-menopausal patients diagnosed with BC is essential. These patients and their relatives who carry the same variant should receive intensive surveillance which includes at least whole-body magnetic resonance imaging (MRI) and central nervous system MRI, according to Toronto protocol^50^. In addition, breast MRI should be offered annually from age 20 years and mammography annually after age 30 years. For these patients, risk-reducing bilateral (adeno)mastectomy should be discussed. For BC patients, mastectomy should be the preferred option in an attempt to avoid radiotherapy. Nonetheless, radiotherapy should be considered when the risk of locoregional recurrence is high.

In the present study, germline variants in BC susceptibility genes other than *BRCA1/2* and *TP53* were also found in approximately 8% of the BC patients. Among BC clinically actionable genes, *ATM*, *CHEK2* and *PALB2* were the most frequently mutated. This finding is in accordance with reports from a recent study analyzing BC predisposition genes in a large cohort of patients^47^. In this work, the cited genes were associated with high or moderate BC risk with similar effect sizes in European and Asian patients which are ancestries well represented in certain regions of Brazil.

*ATM* was the fifth gene with the highest number of P/LP alterations; no founder mutation was found, but it had the highest number of VUS. The most common variant found in *CHEK2* was c.349A>G, representing almost 1/3 of the P/LP variants in this gene. The protein encoded by this allele was found to be defective in functional tests and is likely to be pathogenic^51^. It was found in men with prostate cancer in Portugal and in women with BC in Europe and Brazil^46,52,53^.

Pathogenic variants in other genes, such as *BARD1* and *RAD51C* were also detected. The c.176_177delAG in *BARD1*, was quite common (0.24%) in the present series and, interestingly, it was also detected in other BC Brazilian patients, as well as in Spanish patients, but was not reported in a recent literature review of studies analyzing *BARD1* as a cancer predisposing gene, mainly comprehending French or white people^46,54,55^.

Biallelic *MUTYH* P/LP variants are associated with an autosomal recessive disorder, characterized by polyposis and increased risk of colorectal carcinoma. However, the cancer risk associated with germline variants in individuals carrying only one *MUTYH* defective allele is controversial. Studies have shown that risks of colorectal cancer for carriers of monoallelic variants in *MUTYH* with a first-degree relative with colorectal cancer are sufficiently high to warrant more intensive screening than for the general population, as a consequence NCCN guidelines propose colonoscopy every five years beginning at age 40 years^56^. Nevertheless, there is no strong evidence of the association of increased BC risk and carriers of monoallelic variants in *MUTYH*^47^. In our cohort, the fourth most commonly mutated gene was *MUTYH* due to the high prevalence of two monoallelic variants: *MUTYH* c.1187G>A and *MUTYH* c.536A>G. Our study, in accordance with the majority of previous studies, confirmed that those variants were not associated with increased BC risk. Thus, although it is a frequent finding in patients undergoing multigene panel testing, a monoallelic *MUTYH* variant should not prompt increased surveillance or risk-reducing strategies for BC^57^.

The additional pathogenic variants uncovered by multigene panel testing appears clinically relevant, albeit it is also unveiling a large number of variants that we are still not able to clearly define and classify, the VUS. We have found 767 distinctive VUS in 46.1% of our patients and 88.5% were missense variants. Studies have found that particularly among racial/ethnic minorities there is an increased likelihood of VUS results compared to women of European ancestry due to limited understanding of the normal spectrum of genetic variation in understudied groups^58^. At present, VUS management in the clinical context is challenging. Although it is typically recommended that patients with VUS are managed based on their personal and family history, rather than on the test result, communicating uncertainty has been shown to have the potential to overwhelm patients and increase their worries. In addition, a higher rate of risk reducing surgery among patients with VUS than among patients with negative results has been reported^59^. In order to overcome the challenge of VUS reclassification, the development and improvement of well represented clinical variants databases, predictive algorithms and in vitro functional assays are urgently needed.

## Conclusion

In summary, the largest nationwide cohort of Brazilian BC patients who underwent multigene panel testing identified that *BRCA1/2* accounted for almost 50% of all P/LP germline variants. The use of a multigene panel test almost doubled the identification of P/LP germline variants in BC predisposition genes other than *BRCA1/2*, as well as increased in 12 times the chance of finding a VUS. In general, the spectrum and frequencies of germline variants in non-*BRCA1/2* genes mirrored those described in the literature, except for *TP53* variants. In our cohort, the third most frequently gene mutated was the *TP53* due to the high number of *TP53* R337H carriers in the South and Southeast region of Brazil. As a consequence, the high prevalence of this *TP53* variant has a significant impact in screening and risk-reducing strategies in Brazil.

## Methods

### Study population

Patients were eligible to participate if they were 18 years of age or older at testing, had a personal diagnosis of BC, and were referred for a commercial multigene cancer panel testing at a College of American Pathology (CAP)–accredited laboratory (Mendelics Análise Genômica S.A., São Paulo, SP, Brazil). Informed consent for clinical testing was obtained by the ordering physician. All patient data, which comprehended age at BC diagnosis or age at testing and region of sample collection, were obtained from clinician-completed test requisition forms. This information was anonymized before analysis and there was no missing information. Case selection was limited to one individual per family. In the instance where multiple individuals from the same family underwent multigene panel, the first family member to undergo panel testing was selected for inclusion in this study. The protocol was approved by the Faculdade de Medicina da Universidade São Paulo (FMUSP) Institutional Review Board.

### Panel composition

A custom targeted NGS panel was chosen at the discretion of the ordering clinician and ranged from 20 to 38 genes. All patients underwent comprehensive germline analysis of 20 genes included on the Mendelics curated BC panel: *AKT1*, *ATM*, *BARD1*, *BLM*, *BRCA1*, *BRCA2*, *BRIP1*, *CDH1*, *CHEK2, FANCC, NBN, NF1, PALB2, PTEN, TP53, RAD51C, RAD51D, STK11, PIK3CA, RECQL*. Other 18 genes could be added in the analysis at the discretion of the attending physician: *APC, CDK4, CDKN2A, EGFR, EPCAM*, *MEN1, MET, MLH1, MSH2, MSH6, MUTYH, NF2, PMS2, POLD1, POLE, RB1, RET, WT1.*

Among those genes, 14 were considered clinically actionable because they are referenced in NCCN management guidelines, are commonly included in diagnostic BC panels and there is evidence to support the discussion of personalized BC risk management strategies for patients who test positive^19^. They were separated in 2 categories: high-penetrant: *BRCA1*, *BRCA2*, *CDH1*, *NF1*, *PALB2*, *PTEN*, *STK11*, and *TP53*; and moderate-penetrant: *ATM*, *BARD1*, *BRIP1*, *CHEK2*, *RAD51C,* and *RAD51D*.

### Sequencing and variant interpretation

Genomic DNA was obtained from a buccal swab or peripheral blood sample using standard methods. DNA Sequencing was performed by high-end Illumina platforms (HiSeq 2500 and HiSeq 4000). Base calling was performed using original Illumina tools (bcl2fastq). Bioinformatics pipeline followed Broad Institute best practices (https://gatk.broadinstitute.org/hc/en-us/sections/360007226651-Best-Practices-Workflows). After alignment to the reference genome GRCh37 / UCSC hg19, low quality and duplicate readings were removed, and variants (SNPs/indels) were detected with GATK HaplotypeCaller. Enrichment and analysis concentrated on the coding sequences, flanking intronic regions (± 20 bp) and other specific genomic regions previously identified to harbor causing variants. Promoters, untranslated regions and other non-coding regions were not analyzed. Exonic deletions and duplications (CNV) were identified using ExomeDepth, an R package that estimates the number of copies by comparing the reading depth for each target with the mean reading depth for the same target from samples genotyped from the same sequenced library. If a CNV was identified, multiplex ligation-dependent probe amplification (MLPA) assay was employed to confirm the finding. The variants were classified according to algorithms based on machine learning developed by Mendelics Análise Genômica S.A and described with a nomenclature compatible with the norms and guidelines of the American College of Medical Genetics and Genomics (ACMG)/Human Genome Variation Society (HGVS). Variants interpreted as pathogenic (P) and likely pathogenic (LP) were considered positive. All variants were evaluated by a medical geneticist or pathologist or certified oncologist. Frequencies were calculated according to the total number of patients tested.

### Brazilian genomic database

Reference control data were obtained from the Mendelics Análise Genômica S.A. database, which contains panel and exome sequencing data from 18,919 Brazilian individuals, sequenced as part of various disease-specific genetic tests, excluding samples from cancer cases. Case–control analysis was performed by variant or pooling P/LP variants to the gene level and comparing the frequency in BC patients relative to Brazilian reference controls.

### Statistical analysis

Patients characteristics and sequencing results were tabulated, with descriptive statistics including medians, means, and standard deviations for continuous data and proportions with 95% confidence interval (CI) for categorical data are presented. A χ^2^ test or Fisher exact test was used to compare proportions among cohorts and *P* values less than 0.05 were considered significant. Continuous variables were compared by t tests or Anova, followed by Bonferroni post-test, as necessary. Odds ratios (OR) and 95% CI were calculated by established methods. Statistical analysis was performed using SPSS Version 16.

### Statement

The authors declare that all methods were carried out in accordance with relevant guidelines and regulations.

### Presentation

Preliminary results of this study have been presented at 2018 ASCO Annual Meeting—J Clin Oncol 36, 2018 (suppl; abstr e13610).

## Supplementary Information

Supplementary Table S1.

Supplementary Table S2.

Supplementary Table S3.
